# Knowledge and attitude toward COVID-19 booster dose among health care professionals in Nepal: a cross-sectional study

**DOI:** 10.1097/MS9.0000000000000434

**Published:** 2023-04-04

**Authors:** Kiran Paudel, Sangam Shah, Sandesh Bhusal, Krishna Dahal, Nikita Bhatta, Saurav Pokhrel, Suman Dahal, Milan Gaihre, Anish Mudvari, Pradip Gyanwali

**Affiliations:** a Institute of Medicine, Tribhuvan University, Maharajgunj, Nepal; b Nepal Health Frontiers, Tokha-5, Kathmandu Nepal; c Maharajgunj Nursing Campus, Institute of Medicine, Tribhuvan University, Maharajgunj, Nepal; d Department of General Surgery, Institute of Medicine, Tribhuvan University, Maharajgunj, Nepal; e Department of Internal Medicine, College of Medical Sciences, Chitwan, Nepal

**Keywords:** attitude, booster dose, COVID-19, health care workers, knowledge, vaccines

## Abstract

Vaccination has become the most effective way to combat the coronavirus disease 2019 (COVID-19) pandemic. As there have been reports of a gradual decline in the protection it offers, many countries have decided to administer booster doses of the COVID-19 vaccine. In Nepal, booster doses have been introduced to frontline health workers as a priority group. Therefore, this study aims to assess the knowledge and attitude of health care professionals toward booster doses of COVID-19 vaccines in Nepal.

**Methods:**

A cross-sectional study was conducted from December 2021 to January 2022 among health care professionals working at public health facilities in Nepal. Multivariable logistic regression was performed to identify predictors that correlate with knowledge and attitude toward COVID-19 booster dose. *P* value less than 0.05 was considered statistically significant.

**Results:**

A total of 300 participants were included in the final analysis. Among the study participants, 68.0% and 78.6% had good knowledge and favorable attitude toward COVID-19 booster dose, respectively. Female health care workers and those who had received a single dose of COVID-19 vaccine had significantly lower odds of having good knowledge of COVID-19 booster dose. Similarly, participants with lower educational levels and those who had received a single dose of COVID-19 vaccination had an unfavorable attitude toward COVID-19 booster dose.

**Conclusion:**

This study showed a satisfactory level of knowledge and attitude of health care professionals toward COVID-19 booster dose in Nepal. Health care professionals’ positive attitude toward COVID-19 booster dose vaccine is key to the patient and community safety. Personalized education and risk communication can aid in improving overall awareness and attitudes toward COVID-19 booster dose in such populations.

## Introduction

HIGHLIGHTS

- In this study, 68.0% and 78.6% of the health care professionals had good knowledge and favorable attitude toward COVID-19 booster dose.
- Among the study variables, gender, participants refusing to booster dose, and the number of vaccine doses received are significantly related to knowledge regarding COVID-19 booster dose. Similarly, educational status, profession, and the number of vaccination dose were associated with attitude level.
- Personalized education and risk communication can aid in improving overall awareness and attitudes toward COVID-19 booster dose in such population.

Coronavirus disease (COVID)-19 is caused by the severe acute respiratory syndrome coronavirus-2, (SARS-CoV-2) which is a rapidly transmitting airborne virus that mainly affects the lungs^1^. Since its outbreak in Wuhan in 2019, it has emerged as a major concern for people and has been declared a global pandemic by the WHO^2^. Many people have lost their lives and countries have suffered a major economic downfall. With more than six million deaths worldwide, it has emerged as a major threat to countries and the health care system^3^. With no absolute treatment, physical distancing, proper use of masks, and chemical disinfectants are the only way we could surpass the emerging rise of the COVID-19 virus^4^,^5^. Especially in lower middle-income countries like Nepal, tackling the pandemic has been a major challenge. Over one million people have been infected and 12 thousand people have lost their lives till now in Nepal^3^. With the rapid surge of cases due to new variants, the Epidemiology and Disease Control Division (EDCD) has overtaken the mass vaccination campaign as a major weapon to fight the pandemic.

The mass vaccination campaign started back on 27 January 2021 in Nepal with the aid of one million doses of AstraZeneca (Covishield) provided by India^5^,^6^. Health professionals, elderly people, and prisoners were listed as priority groups. Vaccines like Sinopharm’s Vero Cell (BBIBP-CorV), Pfizer, Moderna, Johnson and Johnson, and AstraZeneca are approved by the Government of Nepal for emergency use^5^,^6^. However, the availability of vaccines does not guarantee their use, it depends on the perception of vaccine efficacy and safety^7^. A study conducted by Subedi *et al*.^8^ in Nepal found that 64.5% of participants thought COVID-19 vaccines were safe and risk-free, and 68.6% agreed that vaccination would be effective in combating the pandemic.

Nepal has been able to provide at least one dose of the vaccine to 53.5% of its general population and a full course of primary vaccination to 42.5% of its total population as of May 2022^9^. Recently, a booster dose of COVID-19 has been introduced to frontline health workers as a priority group. Misconceptions regarding booster dose have surfaced among the general public and health professionals as their safety is still to be guaranteed^10^. Assessing the knowledge and attitude toward vaccination is crucial as vaccine knowledge was one of the strongest factors associated with vaccination intention to receive the COVID-19 vaccine^11^. Health care workers are the frontline soldiers in this pandemic so, the success of the vaccination program in any country depends on their knowledge, awareness, and acceptance of the vaccination process by them. To the best of our knowledge, there has been no prior study among health professionals investing their knowledge and attitude toward booster dose of COVID-19 vaccine in Nepal. Therefore, this study aims to assess the knowledge and attitude of health care professionals toward booster dose of COVID-19 vaccines in Nepal.

## Methods

### Study design, participants, and sampling

A web-based cross-sectional survey was conducted among health care workers in Nepal from December 2021 to January 2022. The study population was the health care workers working at different tiers of health care facilities in Nepal including primary-level health-post to tertiary-level government hospitals and academic institutions/medical colleges. Health care workers included doctors, nurses, pharmacists, diagnostic personnel, paramedics, and public health practitioners. Health care workers aged 18 years or more who agreed to participate in the study were included in the study. Health professionals who have less than 6 months of working experience were excluded from the study.

A convenience sampling procedure was used to recruit the participants.

The sample size was calculated using the formula: *n*=*z* ^2^ *pq*/*e* ^2^ 12. Assuming 70% prevalence (*p*), 5% margin of error (*e*), 95% CI, and 5% nonresponse rate, the final sample size was determined as 338. However, 20 participants declined to participate in the survey and 18 responses were incomplete, therefore, a total of 300 responses were included in the final analysis.

### Measurements

The dependent variable was knowledge and attitude of health care professionals toward COVID-19 booster dose vaccine, while the independent variables were sociodemographic characteristics, health, and COVID-19 vaccine status.

The questionnaire was adopted from the study conducted by Ahmed *et al*.^13^ among health professionals in Ethiopia. We used Bloom’s cutoff point to categorize knowledge (good/poor) and attitude (favorable/unfavorable) of the participants. Knowledge or attitude was considered good/favorable if the score was between 60 and 100% and poor/unfavorable if the score was less than 60%^14^. A total of five questions with responses ‘yes/no’ assessed the participant’s knowledge regarding the COVID-19 booster dose vaccine. Similarly, six questions with responses ‘yes/no’ assessed the participant’s attitude toward COVID-19 booster dose vaccine. Evaluation of knowledge and attitude was done by assigning ‘1’ for a positive response and ‘0’ for a negative response based on a question.

This work is fully compliant with the STROCSS 2021 guideline^15^.

### Data collection

An online Google form, compatible with both computers and smartphones, was used to collect the data from the participants. The questionnaire was administered in the English language through the most commonly used social media platforms in Nepal namely, Facebook, Viber, and WhatsApp. Also, to reach a wider group of health care workers, survey forms were posted in relevant social media groups of them such as ‘Doctor’s Society of Nepal’, ‘Nepalese Nurses’, ‘Public Health Network’, etc.

### Statistical analysis

Data from the Google forms were automatically recorded in Google sheets which was exported to Statistical Package for the Social Sciences (SPSS) version 20 (IBM) for statistical analysis. Descriptive analysis was done by calculating the frequency and percentages of categorical variables. *χ* ^2^-Test was used to determine the association between categorical independent variables and categorical dependent variables. To determine potential factors associated with the outcome variable, multivariable logistic regression using ‘enter’ method was performed, adjusted odds ratio (AOR) and 95% CI were calculated. Those variables with a *P* value less than 0.25 in bivariate analysis were entered into a multivariable logistic regression analysis^16^. For multivariable logistic regression analysis, a *P* value less than 0.05 was considered significantly associated. The variance inflation factor was calculated before fitting into the model for each of the variables which showed no evidence of multicollinearity (<1.5).

### Ethical approval

Ethical approval was obtained from the Institutional Review Committee (IRC) of the Institute of Medicine, Tribhuvan University, Nepal [Reference number: 350^6^–^11^ E^2^ 078/079]. All the participants were informed about the aims and objectives of the study by including the electronic consent form in the Google form questionnaire. Participants gave their consent by ticking the designated box. Confidentiality of the information was maintained thoroughly by de-identification (UIN: researchregistry8649).

## Results

### Sociodemographic characteristics of participants

A total of 300 participants were included in the final analysis. Of them, 55.3% were females and 77.3% were below the age of 30 years. The majority of the participants had completed a bachelor’s degree (71.0%). Brahmin/Chhetri was the major ethnic group (58.0%). Among the participants, 39.3% were doctors, followed by paramedic staff (24.7%), and nursing staff (22.3%). Around 80% of the participants had less than 5 years of working experience (Table 1).

**Table 1:** Sociodemographic characteristics of participants

Characteristics	*N*	%
Sex		
Male	134	44.7
Female	166	55.3
Age		
Less than 30 years	232	77.3
30 years and above	68	22.7
Marital status		
Married	102	34.0
Not married	198	66.0
Educational status		
Diploma	13	4.3
Bachelors	213	71.0
Master and above	74	24.7
Profession		
Doctor	118	39.3
Paramedics	74	24.7
Nurse	67	22.3
Public health	41	13.7
Ethnicity		
Brahmin/Chhetri	174	58.0
Madhesi	60	20.0
Janjati	51	17.0
Others	15	5.0
Working experience		
Less than 5 years	241	80.3
5 years and above	59	19.7

### Participant’s health and COVID-19 vaccination status

A total of 13 (4.3%) respondents had any chronic diseases. About 8.0% of the respondents were at high risk of COVID-19 and 23.7% were at medium risk. A total of 35 (11.7%), 178 (59.3%), and 87 (29.0%) of the participants had taken one, two, and three vaccination doses, respectively. Among them, 134 (44.7%%) participants had some form of side effects postvaccination. Among the participants, 36 (12.0%) participants refused to take the COVID-19 booster dose (Table 2).

**Table 2:** Participant’s health and COVID-19 vaccination status

Characteristics	*N*	%
Have any chronic diseases		
Yes	13	4.3
No	287	95.7
Perceived risk degree in the working area		
High	23	7.6
Medium	71	23.7
Low	206	68.7
Vaccination doses		
One	35	11.7
Two	178	59.3
Three	87	29.0
Any side effects after vaccination		
Yes	134	44.7
No	166	55.3
Have you ever tested positive for COVID-19		
Yes	158	52.7
No	142	47.3
Received any training or orientation about COVID-19		
Yes	52	17.3
No	248	82.7
Do you refuse to get COVID-19 booster dose?		
Yes	36	12.0
No	264	88.0

COVID-19, coronavirus disease 2019.

### Health care worker’s knowledge of COVID-19 booster dose vaccine

Among the participants, 258 (86.0%) knew about the COVID-19 booster dose and 211 (70.3%) knew about the effectiveness of the COVID-19 booster dose. Similarly, 17.7% of the participants perceived that the COVID-19 booster dose increases allergic reactions (Table 3).

**Table 3:** Health care workers’ knowledge of COVID-19 booster dose (*n*=300)

Variables	Category	Frequency (%)
Do you know about the booster dose of COVID-19 vaccine?	Yes	258 (86.0)
	No	42 (14.0)
Do you know about the effectiveness of booster dose of COVID-19 vaccine?	Yes	211 (70.3)
	No	89 (29.7)
Is it very dangerous to health to get a COVID-19 booster dose vaccine?	Yes	101 (33.7)
	No	199 (66.3)
Does COVID-19 booster dose vaccine increase allergic reactions?	Yes	53 (17.7)
	No	247 (82.3)
Does booster dose vaccine increase autoimmune diseases?	Yes	52 (17.3)
	No	248 (82.7)

COVID-19, coronavirus disease 2019.

### Health care worker’s attitude toward COVID-19 booster dose

Out of the total participants, 270 (90%) reported that the booster COVID-19 vaccine dose is safe. Most of the participants 275 (91.7%) stated that they will encourage their families and friends to get vaccinated against COVID-19 and 90.7% were ready to take booster dose without any hesitation. A total of 59% of participants declared that it is not possible to reduce the prevalence of COVID-19 with a booster vaccine dose (Table 4).

**Table 4:** Health care workers’ attitude toward COVID-19 booster dose (*n*=300)

Variables	Category	Frequency (%)
The booster dose of COVID-19 vaccine is safe	Yes	270 (90.0)
	No	30 (10.0)
The booster dose of COVID-19 vaccine is essential for Nepal	Yes	268 (89.3)
	No	32 (10.7)
I will encourage my family, friends, and relatives to get vaccinated against COVID-19	Yes	275 (91.7)
	No	25 (8.3)
I will take a booster COVID-19 vaccine dose without any hesitation if it is available in Nepal	Yes	272 (90.7)
	No	28 (9.3)
It is not possible to reduce the prevalence of COVID-19 without a booster vaccine dose	Yes	177 (59.0)
	No	123 (41.0)
The booster COVID-19 vaccine doses should be distributed fairly to all of us	Yes	257 (85.7)
	No	43 (14.3)

COVID-19, coronavirus disease 2019.

### Factors associated with knowledge and attitude of health care workers toward COVID-19 booster dose

Table 5 presents the factors associated with the knowledge and attitude of health care professionals toward COVID-19 booster dose vaccine. Among the study variables, gender, participants refusing to take booster dose, and the number of vaccine doses received are significantly related to knowledge regarding COVID-19 booster dose. Females (AOR: 0.5, 95% CI: 0.3–0.9) and participants receiving just a single vaccine dose of COVID-19 (AOR: 0.3, 95% CI: 0.1–0.6) had lower odds of having good knowledge regarding COVID-19 booster than males and participants receiving two or three doses of vaccine. Participants not refusing to take COVID-19 booster dose had higher odds of having good knowledge than those who refused (AOR: 2.5, 95% CI: 1.2–5.3).

**Table 5:** Factors associated with knowledge and attitude toward COVID-19 booster dose

	Knowledge			Attitude		
Variable	Good	Poor	Adjusted odds ratio (95% CI)	Favorable	Unfavorable	Adjusted odds ratio (95% CI)
Sex						
Male	99 (73.9)	35 (26.1)	Ref	97 (78.2)	27 (21.8)	Ref
Female	105 (63.3)	61 (36.7)	0.5 (0.3–0.9)*	139 (78.9)	37 (21.1)	1.4 (0.7–2.8)
Age in years						
Less than 30	155 (66.8)	77 (33.2)	–	188 (81.0)	44 (19.0)	2.0 (0.7–5.7)
30 and above	49 (72.1)	19 (27.9)	–	48 (70.6)	20 (29.4)	Ref
Marital status						
Married	69 (67.6)	33 (32.4)	–	76 (74.5)	26 (25.5)	0.7 (0.3–1.7)
Unmarried	135 (68.2)	63 (31.8)	–	160 (80.8)	38 (19.2)	Ref
Educational status						
Diploma	7 (53.8)	6 (46.2)	0.4 (0.1–1.3)	8 (61.5)	5 (38.5)	0.1 (0.03–0.6)*
Bachelors	142 (66.7)	71 (33.3)	0.7 (0.4–1.4)	173 (81.2)	40 (18.8)	0.7 (0.3–1.6)
Masters and above	55 (74.3)	19 (25.7)	Ref	55 (74.3)	19 (25.7)	Ref
Ethnicity						
Bhramin/Chheteri	125 (71.8)	49 (28.2)	1.1 (0.3–4.0)	141 (81.0)	33 (19.0)	1.8 (0.5–6.6)
Madheshi	35 (58.3)	25 (41.7)	0.6 (0.2–2.4)	41 (68.3)	19 (31.7)	0.8 (0.2–3.1)
Janjati	33 (64.7)	18 (35.3)	1.1 (0.2–4.2)	43 (84.3)	8 (15.7)	3.1 (0.7–14.1)
Others	11 (73.3)	4 (26.7)	Ref	11 (73.3)	4 (26.7)	Ref
Profession						
Doctor	88 (74.6)	30 (25.4)	1.8 (0.8–4.0)	88 (74.6)	30 (25.4)	1.9 (0.8–4.5)
Paramedic	45 (60.8)	29 (39.2)	0.9 (0.3–2.1)	65 (87.8)	9 (12.2)	4.6 (1.6–12.8)*
Nurse	44 (65.7)	23 (34.3)	1.3 (0.5–3.5)	56 (83.6)	11 (16.4)	2.2 (0.8–6.5)
Public health	27 (65.9)	14 (34.1)	Ref	27 (65.9)	14 (34.1)	Ref
Refused to get booster dose						
Yes	18 (50)	18 (50)	Ref	24 (66.7)	12 (33.3)	Ref
No	186 (70.5)	78 (29.5)	2.5 (1.2–5.3)*	212 (80.3)	52 (19.7)	1.6 (0.7–3.7)
Number of vaccine doses received						
One	15 (42.9)	20 (57.1)	0.3 (0.1–0.6)*	22 (62.9)	13 (37.1)	0.4 (0.2–0.8)*
Two	121 (68)	57 (32)	Ref	142 (79.8)	36 (20.2)	Ref
Three	68 (78.2)	19 (21.8)	1.7 (0.9–3.2)	72 (82.8)	15 (17.2)	1.2 (0.7–2.5)

COVID-19, coronavirus disease 2019.

Similarly, educational status, profession, and the number of vaccine doses received are significantly associated with the attitude toward COVID-19 booster dose. Participants who received a single COVID-19 vaccine dose (AOR: 0.4, 95% CI: 0.2–0.8) and diploma degree (AOR: 0.1, 95% CI: 0.03–0.6) are less likely to have a favorable attitude than those who received two/three doses of vaccine and have completed master’s degree respectively. The paramedics’ staff are more likely to have a favorable attitude than public health staff (AOR: 4.6, 95% CI: 1.6–12.8) (Table 5).

## Discussion

The availability and efficacy of the COVID-19 booster dose vaccine are critical to the management of COVID-19 pandemic. The perceptions and attitudes of HCWs toward booster dose vaccination significantly impact the public’s attitude toward it. Our study assessed Nepalese health care professionals’ knowledge and attitudes toward the COVID-19 booster dose vaccine.

This study found that 68.0% of health care workers had a good knowledge of the COVID-19 booster dose vaccine. A study conducted in UAE demonstrated that around 55.0% of people involved in the health care profession demonstrated good knowledge of COVID-19 booster dose vaccine which was significantly higher in comparison to other professions^17^. In a study conducted among the general population, only about 31% of participants demonstrated good knowledge about the booster dose^18^. This is not surprising as health care workers are likely to have more exposure to information and education about the vaccine due to their professional training and daily work environment.

In this study, awareness of the COVID-19 booster vaccination dose was substantially linked with the gender with females being less likely (AOR: 0.5, 95% CI: 0.3–0.9) to have good knowledge of COVID-19 booster dose as compared to their counterparts. A study conducted among Indian and Saudi Arabian health care workers found a substantial gender difference in willingness to take COVID-19 booster dose, with more male health care workers reporting that they would be vaccinated as compared with females^19^. Many factors including differences in access to information, socioeconomic status, cultural beliefs, or health-seeking behaviors could have contributed to this disparity.

Unsurprisingly, health care personnel who did not refuse the COVID-19 booster vaccine had 2.5 times higher odds of having good knowledge than those who refused. It is probable that health care workers who had good knowledge about booster dose vaccination were aware of the risk of contracting COVID-19 and wanted to acquire a booster dose as soon as possible for self-protection^20^. Similarly, health care professionals who had only one dose of COVID-19 vaccination were less likely (AOR: 0.3, 95% CI: 0.1–0.6) to have good knowledge than those who had two or three doses of vaccine. This is comparable to the findings of a study conducted among Saudi Arabian health care workers where booster dose acceptance was associated with receiving multiple dose of COVID-19 vaccine^21^.

In this study, 78.6% of the respondents have a favorable attitude toward COVID-19 booster dose. A study conducted among US health care workers found that nearly two-thirds of the respondents were concerned that current vaccines may have decreased efficacy against new variants of COVID-19 and booster doses may be required to provide extra protection^22^. Similarly, findings of a study conducted by Vellappally *et al*.^19^ showed 84% of Indian and 67% of Saudi Arabian health care workers were willing to receive the COVID-19 booster vaccination which denotes a positive attitude of health care workers toward booster dose. These differences in proportion may be due to the differences in COVID-19 prevalence, the availability of COVID-19 vaccines, and vaccine policies in the countries. Concerns over vaccine safety, mistrust of authorities, and concerns about probable long-term side effects could have played a significant role in shaping participants’ attitudes toward booster dose vaccines^19^,^22^.

Educational status was found to be a major determinant in attitudes regarding a COVID-19 booster vaccination dose. In comparison to professionals having Master’s and above degree, professionals with a diploma were less likely to have a favorable attitude toward COVID-19 booster dose (AOR: 0.1, 95% CI: 0.03–0.6). This finding is supported by multiple studies conducted around the world^23^,^24^. Respondents with a higher level of education may be more likely to receive vaccine information, more likely to trust the safety and efficacy of vaccines or may have a better understanding of vaccine science.

Among health care providers, paramedics were more likely to have a favorable attitude toward the COVID-19 booster dose. When compared to public health professionals, paramedics professionals had a 4.6-fold more favorable attitude toward the COVID-19 booster dose. The most likely explanation could be that paramedic health professionals worked in laboratories collecting samples of COVID-19 (nasopharyngeal swabs) and radiological departments and therefore at more risk of contracting COVID-19 due to close contact with the cases^25^,^26^.

In this study, health care personnel who had received a single dose of COVID-19 vaccination had 0.4 times lower odds of having a favorable attitude compared with those who had administered two or three doses of COVID-19 vaccine (AOR: 0.4, 95% CI: 0.2–0.8). This suggests that receiving multiple doses of the vaccine may be associated with a more positive attitude toward COVID-19 booster dose vaccination among health care personnel. This may be due to increased confidence in the vaccine’s efficacy after receiving multiple doses, as well as a reduced perception of side effects.

To the best of the authors’ knowledge, this is the first study to assess health care workers’ knowledge and attitudes concerning the COVID-19 booster dose vaccine in Nepal. However, certain limitations to this study should be acknowledged. This study followed a cross-sectional design that limits our ability to infer causal relationships. As the sample was chosen conveniently, that can potentially skew the results and limit the generalizability of the findings. Similarly, online survey distribution might have overlooked persons in older age groups or in areas without reliable internet access.

## Conclusion

Among the total participants, 68.0% and 78.6% had good knowledge and favorable attitude toward COVID-19 booster dose, respectively. In this study, factors like education, occupation, and the number of vaccination doses received were found as determinants of attitude toward the COVID-19 booster dose. Based on these findings, effective health education and awareness programs might be needed that are aimed at mobilizing and improving COVID-19 booster dose-related knowledge and attitude. Future studies should explore the strategies that can help alleviate the concerns of health care workers regarding the emergence of new variants and the use of vaccines to tackle them.

## Consent

Informed consent was obtained from all study participants to allow the use of anonymous personal and clinical data in research. Confidentiality of the information was maintained thoroughly by de-identification.

## Sources of funding

No funding was received for the study.

## Conflicts of interest disclosure

Authors have no conflict of interest to declare.

## Provenance and peer review

Not commissioned, externally peer-reviewed.

## References

[1] Al-Zalfawi SM Rabbani SI Asdaq SMB . Public knowledge, attitude, and perception towards COVID-19 vaccination in Saudi Arabia. Int J Environ Res Public Health 2021;18:10081. 34639382 10.3390/ijerph181910081 PMC8508088

[2] Livingston E Bucher K Rekito A . Coronavirus disease 2019 and influenza 2019-2020. J Am Med Assoc 2020;323:1122. 10.1001/jama.2020.2633 32207769

[3] Worldometer. Nepal COVID – Coronavirus Statistics. Accessed 22 January 2022. https://www.worldometers.info/coronavirus/country/nepal/

[4] Dolgin E . COVID vaccine immunity is waning – how much does that matter? Nature 2021;597:606–607. 34548661 10.1038/d41586-021-02532-4

[5] Sah R Shrestha S Mehta R . AZD1222 (Covishield) vaccination for COVID-19: experiences, challenges, and solutions in Nepal. Travel Med Infect Dis 2021;40:101989. 33578045 10.1016/j.tmaid.2021.101989 PMC7872846

[6] Nepal’s Vaccination Status. https://kathmandupost.com/health/2021/05/12/nepal-s-vaccination-status

[7] Tamburro M Gaire A Panthee B . COVID-19 vaccine acceptance: a case study from Nepal. COVID 2022 2022;2:1014–1025.

[8] Subedi D Pantha S Subedi S . Perceptions towards COVID-19 vaccines and willingness to vaccinate in Nepal. Vaccines 2021 2021;9:1448. 10.3390/vaccines9121448 PMC8703692 34960194

[9] MoHP. Health Sector Response to COVID-19. 2077;1–5.

[10] Klugar M Riad A Mohanan L . COVID-19 vaccine booster hesitancy (VBH) of healthcare workers in Czechia: National Cross-Sectional Study. Vaccines (Basel) 2021;9:1437. 34960183 10.3390/vaccines9121437 PMC8705445

[11] Wong LP Alias H Danaee M . COVID-19 vaccination intention and vaccine characteristics influencing vaccination acceptance: a global survey of 17 countries. Infect Dis Poverty 2021;10:122. 34620243 10.1186/s40249-021-00900-w PMC8496428

[12] Charan J Biswas T . How to calculate sample size for different study designs in medical research? Indian J Psychol Med 2013;35:121–126. 24049221 10.4103/0253-7176.116232 PMC3775042

[13] Ahmed MH Siraj SS Klein J . Knowledge and attitude towards second COVID-19 vaccine dose among health professionals working at Public Health Facilities in a low income country. Infect Drug Resist 2021;14:3125–3134. 34408455 10.2147/IDR.S327954 PMC8366936

[14] Wang L Abualfoul M Oduor H . A cross-sectional study of knowledge, attitude, and practice toward COVID-19 in solid organ transplant recipients at a transplant center in the United States. Front public Heal 2022;10:880774. 10.3389/fpubh.2022.880774 PMC9539443 36211649

[15] Mathew G Agha R Albrecht J . STROCSS 2021: strengthening the reporting of cohort, cross-sectional and case-control studies in surgery. Int J Surg 2021;96:106165. 34774726 10.1016/j.ijsu.2021.106165

[16] Bursac Z Gauss CH Williams DK . Purposeful selection of variables in logistic regression. Source Code Biol Med 2008;3:17. 19087314 10.1186/1751-0473-3-17 PMC2633005

[17] Jairoun AA Al-Hemyari SS El-Dahiyat F . Assessing public knowledge, attitudes and determinants of third COVID-19 vaccine booster dose acceptance: current scenario and future perspectives. J Pharm policy Pract 2022;15:26. 35346377 10.1186/s40545-022-00422-2 PMC8959269

[18] Abullais SS Arora S Al Shahrani M . Knowledge, perception, and acceptance toward the booster dose of COVID-19 vaccine among patients visiting dental clinics in Aseer region of KSA. Hum Vaccin Immunother 2022;18:2095162. 35856819 10.1080/21645515.2022.2095162 PMC9746421

[19] Vellappally S Naik S Alsadon O . Perception of COVID-19 booster dose vaccine among healthcare workers in India and Saudi Arabia. Int J Environ Res Public Health 2022;19:8942. 35897309 10.3390/ijerph19158942 PMC9332579

[20] Klugar M Riad A Mohanan L . COVID-19 vaccine booster hesitancy (VBH) of healthcare workers in czechia: National cross-sectional study. Vaccines 2021;9:1437. 34960183 10.3390/vaccines9121437 PMC8705445

[21] Alhasan K Aljamaan F Temsah MH . COVID-19 delta variant: perceptions, worries, and vaccine-booster acceptability among healthcare workers. Healthc 2021 2021;9:1566. 10.3390/healthcare9111566 PMC8621199 34828612

[22] Pal S Shekhar R Kottewar S . COVID-19 vaccine hesitancy and attitude toward booster doses among US healthcare workers. Vaccines 2021;9:1358. 34835289 10.3390/vaccines9111358 PMC8617683

[23] Sallam M . COVID-19 Vaccine Hesitancy Worldwide: a concise systematic review of vaccine acceptance rates. Vaccines 2021 2021;9:160. 10.3390/vaccines9020160 PMC7920465 33669441

[24] Biswas N Mustapha T Khubchandani J . The nature and extent of COVID-19 vaccination hesitancy in healthcare workers. J Community Health 2021;46:1244–1251. 33877534 10.1007/s10900-021-00984-3 PMC8056370

[25] Huang L Wang Y Liu J . Short report: factors determining perceived stress among medical staff in radiology departments during the COVID-19 outbreak. Psychol Health Med 2021;26:56–61. 33080151 10.1080/13548506.2020.1837390

[26] Giri LM ☯Paudel K Bhusal S . Perceived stress, stigma, and social support among Nepali health care workers during COVID-19 pandemic: a cross-sectional web-based survey. PLoS Glob Public Heal 2022;2:e0000458. 10.1371/journal.pgph.0000458 PMC10022390 36962228

